# Identification of DYRK1B as a substrate of ERK1/2 and characterisation of the kinase activity of DYRK1B mutants from cancer and metabolic syndrome

**DOI:** 10.1007/s00018-015-2032-x

**Published:** 2015-09-07

**Authors:** Anne L. Ashford, Tom P. J. Dunkley, Mark Cockerill, Rachel A. Rowlinson, Lisa M. Baak, Raffaella Gallo, Kathryn Balmanno, Louise M. Goodwin, Richard A. Ward, Pamela A. Lochhead, Sylvie Guichard, Kevin Hudson, Simon J. Cook

**Affiliations:** 1 Signalling Laboratory, The Babraham Institute, Babraham Research Campus, Cambridge, CB22 3AT UK; 2 AstraZeneca, Alderley Park, Macclesfield, Cheshire, SK10 4TG UK; 3 Present Address: Roche Innovation Center Basel, Basel, Switzerland; 4 Present Address: Paterson Institute for Cancer Research, University of Manchester, Wilmslow Road, Manchester, M20 4BX UK; 5 Present Address: AstraZeneca, Waltham, MA 02451 USA

**Keywords:** DYRK1B, ERK1/2, KRAS, Phosphorylation, Protein kinase, RAF

## Abstract

The dual-specificity tyrosine-phosphorylation-regulated kinase, DYRK1B, is expressed de novo during myogenesis, amplified or mutated in certain cancers and mutated in familial cases of metabolic syndrome. DYRK1B is activated by *cis* auto-phosphorylation on tyrosine-273 (Y273) within the activation loop during translation but few other DYRK1B phosphorylation sites have been characterised to date. Here, we demonstrate that DYRK1B also undergoes *trans*-autophosphorylation on serine-421 (S421) in vitro and in cells and that this site contributes to DYRK1B kinase activity. Whilst a DYRK1B^S421A^ mutant was completely defective for p-S421 in cells, DYRK1B inhibitors caused only a partial loss of p-S421 suggesting the existence of an additional kinase that could also phosphorylate DYRK1B S421. Indeed, a catalytically inactive DYRK1B^D239A^ mutant exhibited very low levels of p-S421 in cells but this was increased by KRAS^G12V^. In addition, selective activation of the RAF-MEK1/2-ERK1/2 signalling pathway rapidly increased p-S421 in cells whereas activation of the stress kinases JNK or p38 could not. S421 resides within a Ser-Pro phosphoacceptor motif that is typical for ERK1/2 and recombinant ERK2 phosphorylated DYRK1B at S421 in vitro. Our results show that DYRK1B is a novel ERK2 substrate, uncovering new links between two kinases involved in cell fate decisions. Finally, we show that DYRK1B mutants that have recently been described in cancer and metabolic syndrome exhibit normal or reduced intrinsic kinase activity.

**Electronic supplementary material:**

The online version of this article (doi:10.1007/s00018-015-2032-x) contains supplementary material, which is available to authorized users.

## Introduction

The dual-specificity tyrosine-phosphorylation-regulated kinases (DYRKs) are an evolutionarily conserved family of protein kinases that are found within the CMGC group (*C*DK, *M*APK, *G*SK and *C*LK families) of the eukaryote kinome [1–4]. The mammalian DYRKs are divided into class I (DYRK1A and DYRK1B) and class II (DYRK2, DYRK3 and DYRK4) based on sequence homologies. The DYRKs require phosphorylation of the second tyrosine in the characteristic tyrosine-X-tyrosine (Y–X–Y) motif within their activation loop for kinase activity [5, 6]. In contrast to many other kinases, this activation loop phosphorylation is not catalysed by an upstream, ‘activating kinase’ but occurs during translation when DYRKs undergo intramolecular *cis*-autophosphorylation on tyrosine [7]. As co-translational activation loop tyrosine phosphorylation is a one-time-only event, it was proposed that once synthesised, the mature DYRKs lose tyrosine kinase activity and phosphorylate substrates exclusively on serine or threonine residues. However, recent studies have demonstrated that mature DYRK1A retains the ability to autophosphorylate on other tyrosine residues outside the activation loop [8]. Regardless, substrate phosphorylation appears to occur exclusively on serine or threonine, usually within a proline-directed context (pS-P or pT-P) [9, 10]. The DYRKs have roles in controlling transcription, mRNA splicing, cell cycle progression, survival and differentiation [2, 3]. For example, loss of a single copy of Dyrk1a in mice leads to increased apoptosis and decreased brain size in mice [11]. In addition, the *DYRK1A* gene is situated in the Down’s syndrome (DS) critical region on chromosome 21 (21q22.1–22.3), is over-expressed in both foetal and adult brains of DS individuals [12] and is thought to contribute to the clinical features of Down’s syndrome [13–15]. Indeed, *Dyrk1a* is the triplicated gene that causes decreased nuclear Cyclin D1 levels and early cortical neurogenic defects in a mouse model of DS [16]. Together, these studies emphasise the importance of *DYRK1A* gene dosage and activity.

As a result of their co-translational activation loop phosphorylation the DYRKs are active once translated, however, there is growing evidence that some DYRKs are subject to additional post-translational modification and regulation. Autophosphorylation of DYRK1A allows binding of 14-3-3, which promotes DYRK1A catalytic activity [17, 18]. In *C. elegans,* the DYRK homologue, minibrain kinase-2, is activated during oocyte maturation by cyclin-dependent kinase-1 (CDK1)-dependent phosphorylation of serine 68, a residue outside of the kinase domain that is required for full activity in vivo [19]. DYRK2 is phosphorylated at T33 and S369 (numbering corresponding to the short form of DYRK2) by the Ataxia telangiectasia mutated kinase (ATM) in response to genotoxic stress; this inhibits the ubiquitination of DYRK2, which can then phosphorylate S46 of the tumour suppressor p53 [20]. Finally, mass spectrometry of human DYRK4 expressed in HEK293 cells has identified phosphorylated Ser and Thr residues, indicating that DYRK4 is phosphorylated by other cellular protein kinases [21].

DYRK1B is implicated in promoting differentiation in several models: for example, it is expressed de novo during myogenesis [22] and undergoes differential splicing during adipogenesis [23]. Indeed, DYRK1B can promote cell cycle arrest by multiple mechanisms including promoting cyclin D1 (CCND1) degradation [24, 25] by direct phosphorylation at T286 [25] and increasing the expression of the cyclin-dependent kinase inhibitors p21^CIP1^ and p27^KIP1^ [24, 25]. Mutations in *DYRK1B* have been reported in an inherited form of metabolic syndrome associated with early-onset coronary artery disease, obesity, hypertension and diabetes [26]. In addition, *DYRK1B* is amplified [27, 28] and mutated [29] in certain cancers and has been reported to promote cell survival [30–32]. Despite this, less is known about the post-translational regulation of DYRK1B. It has been suggested that oncogenic KRAS stimulates DYRK1B kinase activity [33] and that DYRK1B is a downstream effector of KRAS [34] but the molecular details of this regulation remain unclear.

There is growing interest in inhibiting DYRKs and several small molecule inhibitors of the class I DYRKs have been described including DYRK1B-selective inhibitors such as AZ191 [25] and dual 1A/1B inhibitors such as INDY [35] and Harmine [36]. These inhibitors are selective for the Ser/Thr kinase activity of the mature DYRKs [25, 36], only inhibiting the Tyr kinase activity at very high doses, and are, therefore, useful in helping to define DYRK substrates and DYRK functions. To progress our interest in DYRK1B, we sought to identify DYRK1B autophosphorylation sites that were dependent on the Ser/Thr kinase activity of mature DYRK1B, since these might serve as biomarkers for DYRK1B activity and DYRK1B inhibitors. Here, we identify serine-421 (S421) as a site of DYRK1B *trans*-autophosphorylation that is inhibited by DYRK1B Ser/Thr kinase inhibitors and contributes to DYRK1B kinase activity. Remarkably, S421 is also phosphorylated in a MEK1/2-ERK1/2-dependent fashion in cells and by ERK2 in vitro, defining DYRK1B as a new substrate of ERK2 and providing a link between between two kinases involved in cell fate decisions. Finally, we show that mutations in DYRK1B that are found in cancer or metabolic syndrome either inhibit or are without effect on DYRK1B kinase activity. These results provide new insights into the regulation and role of DYRK1B.

## Materials and methods

### Materials

AZ191 (DYRK1B inhibitor) and selumetinib (AZD6244/ARRY-142886, an allosteric MEK1/2 inhibitor) were provided by AstraZeneca, Alderley Park, Macclesfield, UK. Palbociclib (PD-0332991, CDK4/6 inhibtor) and SCH772984 (ERK1/2 inhibitor) were from Selleck. Horseradish peroxidase-conjugated secondary antibodies were from Bio-Rad, and detection was with the enhanced chemiluminescence (ECL) system (GE Healthcare). All other reagents were from Sigma.

### Antibodies

DYRK1B antibodies produced in rabbits using the immunogen CGLRGVPQSTAASS for the Western blot antibody and MAVPPGHGPFSGC for the antibody used for immunoprecipitation assays were provided by AstraZeneca (Alderley Park, Macclesfield, UK) and were described previously. The p-S421-DYRK1B antibody, also from AstraZeneca, was produced in rabbits immunised with the BSA conjugated phospho-peptide ac-CGEPAARI(P)SPLGALQHG-nh2, prepared using standard procedures [37]. Antibodies specific for p-T202/Y204-ERK1/2 (9106), ERK1/2 (4695), p-T180/Y182-p38 (9211), p-T183/Y185-JNK (9251), pT286-cyclin D1 (2921), p-S33/S37/T41-β Catenin (9561) were from Cell Signaling Technologies; ERK1 (610031), HSP90 (610418) and β-Catenin (610153) were from BD Biosciences, β-actin (A5441) was from Sigma; GFP (11814460001) was from Roche; cyclin D1 (CC12) and Ras (OP40) were from Calbiochem. JNK1/3 (sc474) and ERK2 (sc154) were from Santa Cruz Biotechnology; Flag (MAB3118) was from Millipore; p38 antibody was provided by the Babraham Institute Monoclonal Antibody Facility.

### Plasmids

For the mass spectrometry experiments, DYRK1B sequence (Swissprot Q9Y463; Embl AF205861) was ordered from Geneart with a c-terminal myc-tag and subcloned into pcDNA3. pcDNA3-DYRK1B was mutated by site-directed mutagenesis to generate catalytically inactive Y271F/Y273F and K140R constructs. For other experiments DYRK1B was amplified by PCR from pOTB7-DYRK1B (from Mammalian Gene Collection) and subcloned into pCMV-Tag2B and pEGFP-C3. pCMV-Tag2B-DYRK1B was mutated by site-directed mutagenesis to generate to catalytically inactive DYRK1B (D239A). pCGN-KRAS12V was from Adrienne Cox, Department of Pharmacology, University of North Carolina at Chapel Hill. The sequences of all oligos used are available upon request.

### Analysis of in vitro autophosphorylation of recombinant DYRK1B by mass spectrometry

Recombinant DYRK1B expressed in insect cells (Carna Biosciences) was allowed to autophosphorylate in the presence of ATP. The protein was then resolved on a Novex gel followed by Coomassie staining. The Coomassie bands were reduced with DTT (Sigma), alkylated with iodoacetamide (Sigma), followed by digestion with: trypsin only; AspN and trypsin; chymotrypsin only; chymotrypsin and trypsin (all enzymes supplied by Roche). The resultant peptides were extracted and concentrated. The sample was chromatographed using the Dionex U3000 nanoflow chromatography system (ThermoFisher), the outlet flow ran directly into the Qstar Elite mass spectrometer (ABSciex) for analysis via the nanoflow probe at a flow rate of 300 nl/min. A 40 min reversed phase gradient was run using a 300 µm i.d. × 5 mm C18 PepMap (Dionex ThermoFisher) trapping pre-column and a 75 µm i.d. × 15 cm C18 ACE analytical column (Hichrom). The Qstar collected data in positive ion mode and an auto-switching setup was initiated with automatic precursor selection based on peak intensity and charge state. The subsequent data files generated were searched against the Swissprot database, using the Mascot Daemon software. The searches were then manually verified to confirm phosphorylation sites. From the MSMS data of the phosphorylated and non-phosphorylated peptides, the parent mass and key daughter ion masses were used to generate an MRM (multiple reaction monitoring) method on the 4000 Qtrap mass spectrometer (ABSciex).

### Analysis of DYRK1B autophosphorylation in recombinant enzyme and cells by mass spectrometry using MRM

Recombinant DYRK1B enzyme was incubated with ATP in the presence or absence of 10 μM AZ’294 (an early DYRK1B HTS hit, with an IC_50_ of < 0.03 μM in an in vitro enzyme assay), and run on a Novex gel followed by Coomassie Staining. Reduction, alkylation and digestion with trypsin was carried out as above. Wild-type or mutant myc-DYRK1B proteins were transiently expressed in COS-1 cells. DYRK1B proteins were enriched from whole cell lysates using anti-myc antibody, after treatment with 10 μM AZ’294 or 0.1 % DMSO for 2 h. Immuno-complexes were digested with trypsin prior to nano-liquid chromatography mass spectrometry. MRM was performed on the 4000 Qtrap mass spectrometer using the previously generated method from the recombinant analysis. The sample was chromatographed using the Dionex U3000 nanoflow chromatography system (ThermoFisher) the outlet flow ran directly into the 4000 Qtrap for analysis via the nanoflow probe at a flow rate of 700 nl/min. A 5 min reversed phase gradient was run using a 300 µm i.d. × 5 mm C18 PepMap (Dionex ThermoFisher) trapping pre-column and a 75 µm i.d. × 15 cm C18 ACE analytical column. The column was then equilibrated culminating in a 12.5 min run time per sample. The non-phosphorylated and phosphorylated masses of the pS421 containing tryptic peptide were monitored. Peak quantification was established using ABSciex software, this was manually verified and exported to Excel for further data analysis.

### Cells and cell culture

Cell culture reagents were purchased from Invitrogen. HEK293 cells were maintained in DMEM supplemented with 10 % FBS, 2 mM l-glutamine, 100 Uml^−1^ penicillin, 0.1 μgml^−1^ streptomycin. HR1 and HM3 cells were maintained in HEK293 media supplemented with G418 equivalent to 400 μgml^−1^ neomycin (HR1) or 2 μgml^−1^ Puromycin (HM3) to maintain stable clone selection. HD1B cells were maintained in HEK293 media supplemented with 10µgml^−1^ Blasticidin, 100 µgml^−1^ Zeocin and G418 equivalent to 400 μgml^−1^ neomycin. Cells were routinely passaged before 80 % confluency.

### Preparation of cell extracts and Western blotting

Cells were lysed in ice-cold TG lysis buffer, assayed for protein content and fractionated by SDS-PAGE as described previously [38]. SDS-PAGE gels were transferred to Immobilon P membranes (Millipore), which were blocked in 0.1 % (v/v) Tween-20–TBS (tris-buffered saline) containing 5 % (w/v) powdered milk and probed with the indicated antibodies. Immune complexes were visualised with the ECL system (GE Healthcare).

### In vitro DYRK kinase assay

FLAG-DYRK1B [wild-type (WT), kinase-dead (KD, D239A) or S421A (SA)] were immunoprecipitated from whole cell lysates with anti-FLAG antibodies and then assayed for kinase activity by incubating with 50 μM Woodtide with two additional lysines attached to the *N* terminus to allow it to bind to P81 paper (KKISGRLSPIMTEQ), 50 mM Tris/HCl, pH 7.5, 0.1 mM EGTA, 0.1 % (v/v) 2-mercaptoethanol, 10 mM MgCl_2_, 0.1 mM [γ-^32^P]ATP in a total volume of 50 µl for 20 min at 30 °C as described previously [7]. For each experiment, a single IP was used to generate three technical replicates of ^32^P incorporation in the in vitro assay in addition to quantifying the amount of DYRK1B present by immunoblot. In other experiments (Fig. 2b) FLAG-DYRK1B (kinase-dead; D239A or K140 M) was immunoprecipitated from whole cell lysates with anti-FLAG antibodies and used as a susbstrate for GST-DYRK1B (Full length recombinant human DYRK1B expressed in insect cells, Invitrogen PV4649). Each 50 µl reaction contained 0.3 µg GST-DYRK1B and 10 µl kinase-dead DYRK1B substrate beads in a buffer containing 50 mM Tris/HCl, pH 7.5, 0.1 mM EGTA, 0.1 % (v/v) 2-mercaptoethanol, 10 mM MgCl_2_, 0.1 mM ATP. Reactions were incubated at 30 °C for 50 min and terminated by the addition of 20 µl 4 × Laemmli Buffer and heating at 95 °C for 5 min. Reactions were fractionated by SDS-PAGE, transferred onto PVDF membrane and immunoblotted with the indicated antibodies.

### In vitro ERK2 kinase assay

FLAG-DYRK1B (WT or S421A) or empty vector was expressed in HEK293 cells for 24 h in the presence of 10 µM AZ191. FLAG-DYRK1B was isolated from whole cell extracts by anti-flag immunoprecipitation and used as a substrate in an ERK2 in vitro kinase assay. Each 50 µl reaction contained 0.4 µg GST-ERK2 (Sigma-Aldrich, E1283), 20 µl substrate beads and 50 µM [γ-^32^P]ATP (2.5 µCi per reaction) in a buffer containing 7 mM MOPS (pH 7.2), 3.5 mM glycerol 2-phosphate, 7 mM MgCl_2_, 1.4 mM EGTA, 0.56 mM EDTA and 0.07 mM DTT. Reactions were incubated at 30 °C for 1 h and terminated by the addition of 17 µl 4× Laemmli Buffer and heating at 95 °C for 5 min. Reactions were separated by SDS-PAGE, transferred onto PVDF membrane and ^32^P incorporation was detected by autorad followed by probing with the indicated antibodies.

### DYRK1B siRNA

DYRK1B siRNA (Thermo siGENOME, MQ-004806-01-0002) and GFP siRNA (Qiagen 1027310) were dissolved in nuclease-free water to generate stock solutions of 20 µM and used at a final concentration of 30 nM. For each well of a six well plate, 5 µl of Lipofectamine 2000 was diluted in 250 µl OptiMEM and incubated at room temperature for 5 min. siRNA were diluted in 250 µl OptiMEM prior to the addition of 250 µl of the Lipofectamine-OptiMEM solution. The transfection mixes were incubated at room temperature for 20 min to allow the formation of siRNA complexes. Cells were seeded the previous day in Pen/Strep-free medium to achieve a confluency of 50 % on the day of transfection. 500 µl of the transfection solution was added to each well of the tissue culture plate and the cells were incubated 37 °C in a humidified incubator with 5 % (v/v) CO2 for 16 h. After this 16 h, the medium was changed to fresh Pen/Strep-free media containing DMSO or 1 µM Selumetinib. The cells were incubated for a further 48 h at 37 °C in a humidified incubator with 5 % (v/v) CO2 prior to harvesting.

### Analysis of cell cycle distribution and cell death

Cell cycle distribution (G_1_, S, G_2_/M) and the fraction of dead cells (those with sub-G1 DNA content) was assessed by staining fixed cells with propidium iodide followed by flow cytometry as described previously [38, 39].

## Results

### Identification of S421 as a novel DYRK1B autophosphorylation site that promotes kinase activity

**Fig. 1:**
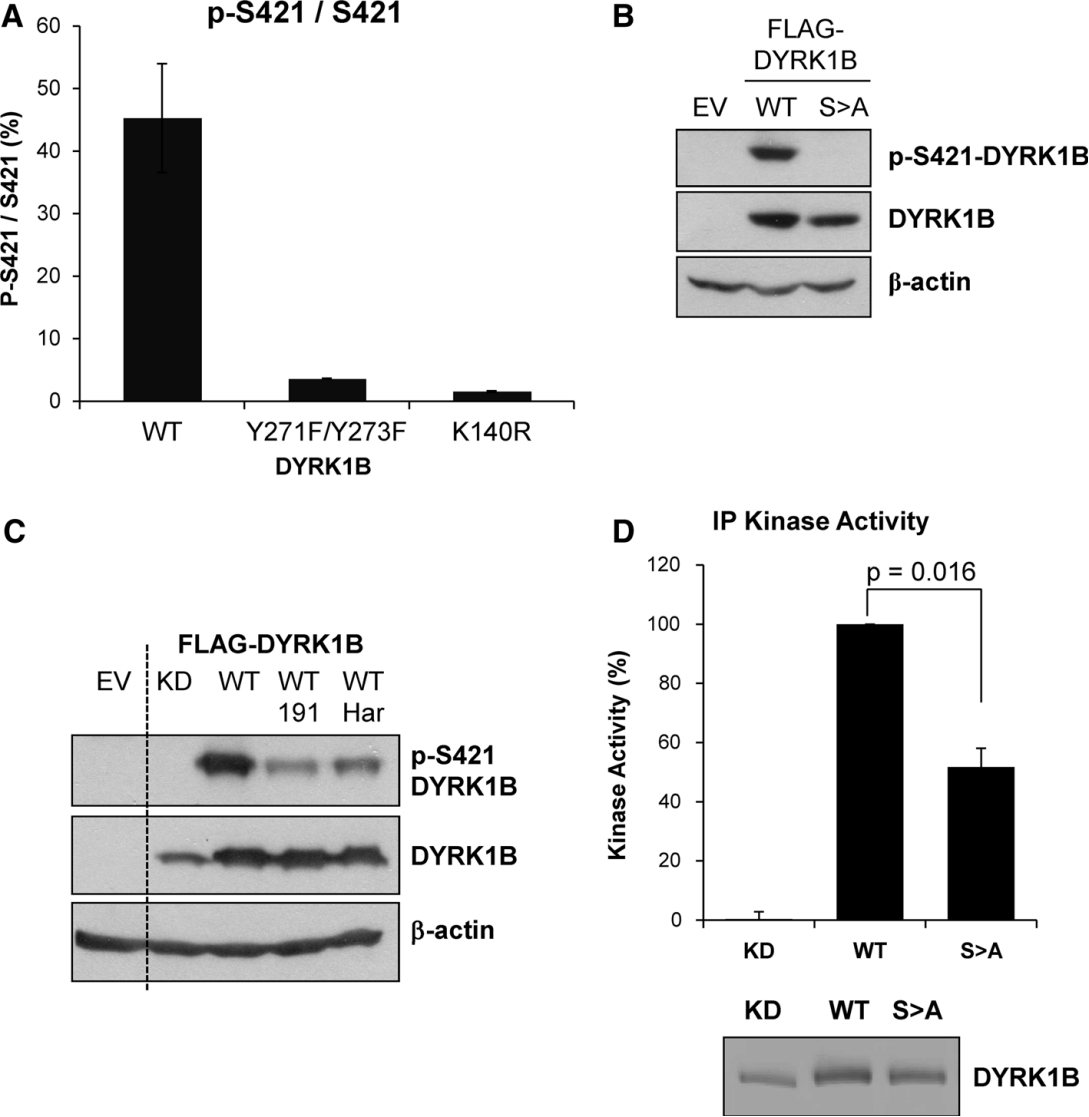
S421 is a site of DYRK1B autophosphorylation that contributes to kinase activity. **a** Wild-type or mutant myc-DYRK1B proteins were expressed in COS-1 cells. DYRK1B proteins were enriched from whole cell lysates using anti-myc antibody. Immuno-complexes were digested with trypsin prior to nanoLC-mass spectrometry analysis for relative phosphorylation of pS421. *Error bars* show standard deviation (*N* = 2). **b** Empty Vector (EV), Wild-type FLAG-DYRK1B (WT) or FLAG-DYRK1B^S421A^ (SA) were transiently co-expressed in HEK293 cells for 24 h. Whole cell extracts were separated by SDS-PAGE, transferred onto PVDF membrane and immuno-blotted with the antibodies indicated. **c** Empty vector (EV), kinase-dead (KD, D239A) or wild-type (WT) FLAG-DYRK1B were transiently co-expressed in HEK293 cells. 6 h post-transfection, the cells were treated with vehicle control, 1 µM AZ191 or 1 µM harmine for a further 24 h. Whole cell extracts were separated by SDS-PAGE, transferred onto PVDF membrane and immuno-blotted with the antibodies indicated. Data in b+c are from single experiments representative of five separate experiments. **d** FLAG-DYRK1B WT, kinase-dead (KD, D239A) or S421A proteins were transiently expressed in HEK293 cells for 24 h and immuno-precipitated from whole cell extracts with an anti-DYRK1B antibody. The resultant immuno-complexes were divided into two aliquots. The first aliquot was assayed for kinase activity against the synthetic substrate peptide Woodtide (50 μM) (*upper panel*). The second aliquot was separated by SDS-PAGE gels and transferred onto PVDF membrane. The amount of DYRK1B protein in each sample was determined by quantitative analysis of the immunoblot using Licor Odyssey (*lower panel*). DYRK1B kinase activity levels were normalised to the amount of DYRK1B present in each immuno-precipitate. Data were averaged from five separate experiments each with three technical replicates with each bar representing the mean ± standard deviation. Statistical analysis was performed using a two-tailed paired *t* test

When expressed in cells catalytically inactive DYRK1B exhibits enhanced migration on SDS-PAGE gels compared to wild-type suggesting that DYRK1B normally undergoes autophosphorylation in vivo [25]. Activation of DYRK1B requires auto-phosphorylation in *cis* at tyrosine 273 (Y273) within the activation loop. However, once translation is complete mature DYRK1B phosphorylates substrates on Ser/Thr residues in *trans*. We wished to develop an assay to monitor the kinase activity of mature DYRK1B in cells, hoping that this might serve as a biomarker to support the development of DYRK1B inhibitors. To determine if DYRK1B could autophosphorylate on other residues, we analysed potential auto-phosphorylation sites on recombinant DYRK1B by proteolytic digestion and mass spectrometry (MS). Using a combination of endoproteases we achieved 85 % coverage of DYRK1B and identified twelve phosphorylation sites including p-Y63 and p-Y273 that have been previously reported (Supplementary Table 1). A targeted MS method was developed to quantify these phosphorylation sites in Cos1 cells expressing Myc-DYRK1B using multiple reaction monitoring (MRM). This analysis identified four phosphorylation sites in cells but only one, S421 in the peptide IpSPLGALQHGFFR, was inhibited when cells were incubated in the presence of AZ’294, an early stage precursor of the DYRK1B inhibitor AZ191 [25] (Table 1). Cellular p-S421 was almost completely abolished when catalytically inactive DYRK1B mutants were expressed in Cos1 cells, including the activation loop mutant DYRK1B^Y271F/Y273F^ or the catalytic lysine mutant DYRK1B^K140R^ (kinase-dead or KD); these results demonstrated that p-S421 in cells was strongly dependent on DYRK1B kinase activity (Fig. 1a).

**Table 1:** Phosphorylation sites detected by mass spectrometry on both recombinant and cellular DYRK1B and the effect of a DYRK1B inhibitor on these sites

Phosphorylation site	In vitro enzyme		In cell enzyme	
	Phospho-peptide detected?^a^	Modulated by DYRK inhibitor?^b^	Phospho-peptide detected?^b^	Modulated by DYRK inhibitor?^b^
S49 KL**pS**VDLIK	Yes	Yes	Yes	No^c^
Y273 IYQ**pY**IQSR	Yes	No	Yes	No^c^
S421 I**pS**PLGALQHGFFR	Yes	Yes	Yes	Yes^c^
S531/533/534 THQAPA**S**A**SS**LPGTGAQLPPQPR	Yes	Yes	Yes	No^c^

^a^Data from initial phospho-peptide mapping of recombinant DYRK1B undergoing autophosphorylation in vitro

^b^Multiple reaction monitoring (MRM) analysis

^c^The non-phosphorylated peptides corresponding to pS49, pY273, pS421 and pS531/533/534 were detected in the untreated cells

To progress this analysis further a p-S421 phospho-specific antibody was raised in rabbits and validated by its ability to detect wild-type DYRK1B but not the non-phosphorylatable DYRK1B^S421A^ mutant when they were both expressed in HEK293 cells (Fig. 1b). Treatment of HEK293 cells with either of two DYRK1B inhibitors, AZ191 [25] or harmine [36], strongly inhibited p-S421 demonstrating that DYRK1B kinase activity was required for efficient S421 phosphorylation in cells (Fig. 1c). In contrast, we have previously shown that these inhibitors do not inhibit auto-phosphorylation at tyrosine, representing p-Y273 and p-Y63 [25], demonstrating that at the doses used they selectively inhibited the Ser/Thr kinase activity of mature DYRK1B.

Since S421 is located in the DYRK1B kinase domain we determined if S421 phosphorylation had any role in DYRK1B kinase activity by comparing wild-type and DYRK1B^S421A^ in kinase assays. DYRK1B proteins were expressed in HEK293 cells and isolated by immunoprecipitation prior to being assayed for kinase activity in vitro; kinase activity was normalised to the amount of wild-type or mutant DYRK1B that was recovered in the immunoprecipitation. These assays revealed that DYRK1B^S421A^ exhibited a significant 50 % reduction in intrinsic kinase activity compared to wild-type DYRK1B (Fig. 1d) whereas kinase-dead DYRK1B^D239A^ (KD) was completely defective. However, since only ~50 % of DYRK1B was phosphorylated at S421 when over-expressed (Fig. 1a), this is probably an underestimate of the contribution of S421 to DYRK1B kinase activity. Thus, S421 phosphorylation contributes to DYRK1B kinase activity.

### DYRK1B autophosphorylation at S421 occurs in *trans*

**Fig. 2:**
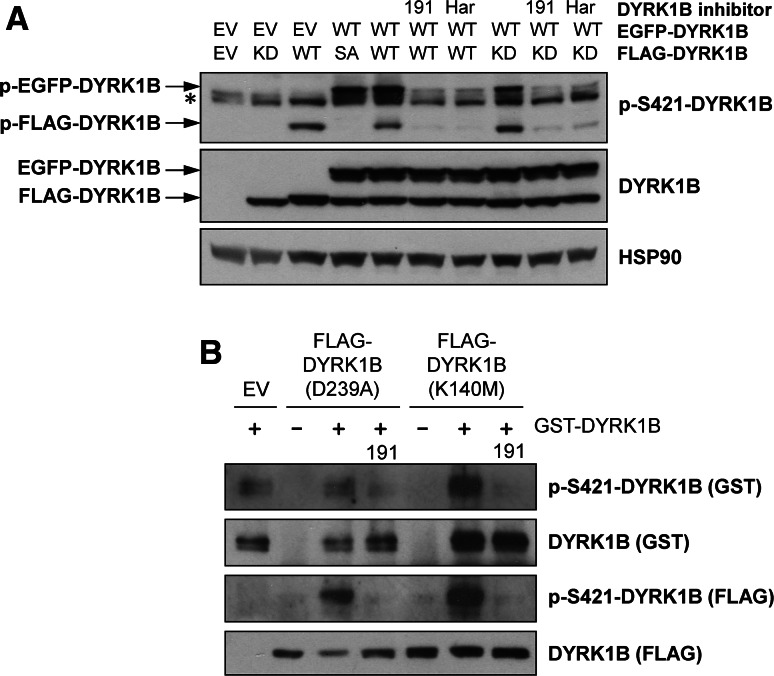
S421 autophosphorylation of DYRK1B occurs *in trans*. **a** Wild-type EGFP-DYRK1B was transiently co-expressed in HEK293 cells along with wild-type (WT), kinase-dead (KD, D239A) or S421A (SA) FLAG-DYRK1B. 6 h post-transfection, cells were treated with 1 µM AZ191, 1 µM harmine or vehicle control for a further 24 h. Whole cell extracts were separated by SDS-PAGE, transferred onto PVDF membrane and immuno-blotted with the specified antibodies. All data are taken from a single experiment representative of three replicate experiments. The asterisk indicates the position of a non-specific band. **b** Empty Vector (EV) or kinase-dead DYRK1B (K140M or D239A) were transfected into HEK293 cells. 24 h post-transfection, DYRK1B proteins were isolated by anti-FLAG immunoprecipitation and subsequently used as a substrate for recombinant GST-DYRK1B (wild-type) in an in vitro kinase assay as describe in “Materials and methods”. Terminated assay reactions were separated by SDS-PAGE and immunoblotted with the indicated antibodies. Data shown are from a single experiment representative of three separate experiments with similar results

Based on the current model of DYRK activation [4, 7], DYRK1B autophosphorylation of Y273 is thought to occur in *cis* through an intramolecular mechanism. To investigate if S421 auto-phosphorylation also occurred in *cis*, we expressed discrete DYRK1B proteins of different sizes (using either EGFP-DYRK1B or FLAG-DYRK1B) so that we could distinguish S421 phosphorylation in *cis* or in *trans* (Fig. 2a). FLAG-DYRK1B^D239A^ (KD) expressed alone was not phosphorylated on S421, nor was wild-type EGFP-DYRK1B able to phosphorylate FLAG-DYRK1B^S421A^ (SA). However, both EGFP- and FLAG-tagged wild-type DYRK1B auto-phosphorylated on S421, and this was blocked by AZ191 or harmine. Finally, FLAG-DYRK1B^D239A^ (KD) was phosphorylated on S421 when co-expressed with wild-type EGFP-DYRK1B and this was blocked by AZ191 or harmine, suggesting this was an intermolecular phosphorylation (Fig. 2a). In addition, we expressed two different kinase-dead DYRK1B mutants—DYRK1B^K140M^ or DYRK1B^D239A^—in HEK293 cells, immunopurified them and then incubated them in vitro with purified recombinant GST-DYRK1B; this is a far simpler system than co-expression in cells (Fig. 2a). These experiments demonstrated that purified GST-DYRK1B could indeed phosphorylate the two different kinase-dead DYRK1B mutants in vitro (Fig. 2b) and in both cases this in vitro trans-autophosphorylation was abolished by AZ191. Whilst we cannot rule out the potential involvement of an accessory protein co-purifying with the DYRK1B mutants, these results strongly suggest that one DYRK1B molecule can directly phosphorylate another on S421 in vitro. Recombinant GST-DYRK1B was also able to autophosphorylate at S421 under these conditions and this was again inhibited by AZ191. In summary, these data strongly suggest that S421 is a novel site of DYRK1B auto-phosphorylation that can proceed through an *in trans* mechanism.

### Activation of the RAF-MEK-ERK1/2 signalling pathway also promotes S421 phosphorylation in cells

**Fig. 3:**
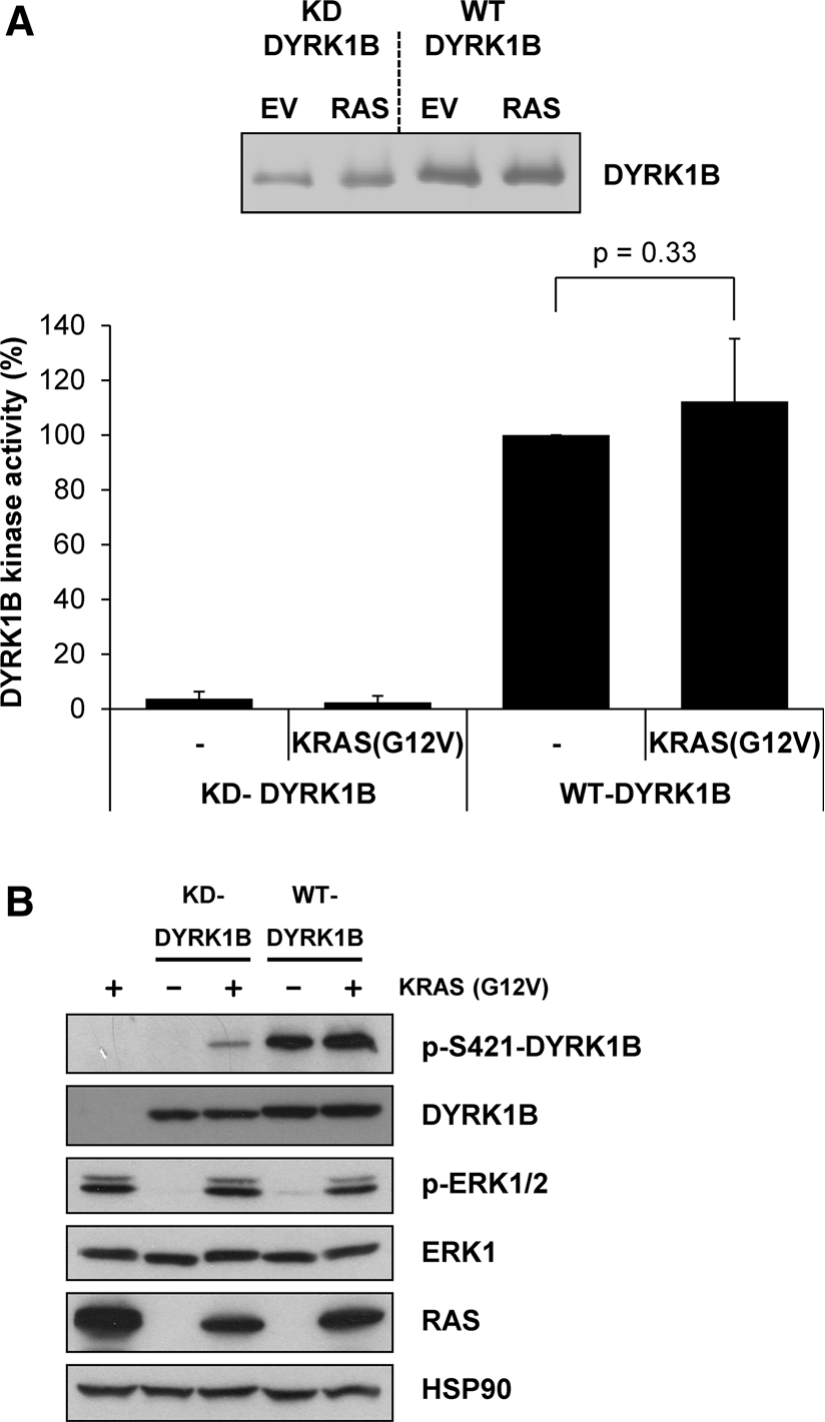
KRAS^G12V^ promotes S421 phosphorylation. Wild-type or kinase-dead DYRK1B were transiently expressed together with empty vector or KRAS^G12V^ for 24 h in HEK293 cells. **a** FLAG-DYRK1B proteins were isolated from whole cell extracts by immunoprecipitation and immuno-complexes were divided into two aliquots. The first aliquot was separated by SDS-PAGE, transferred onto PVDF membrane and immunoblotted for DYRK1B. DYRK1B levels were quantified on Licor Odyssey (*upper panel*). Data are from a single experiment representative of five separate experiments. The second aliquot was incubated with the synthetic DYRK substrate peptide Woodtide in an in vitro kinase assay to determine kinase activity. In vitro kinase activity was normalised to the amount of DYRK1B proteins present on the immuno-blot (*lower panel*). Data in the graph are presented as mean ± standard deviation from five separate experiments each with three technical replicates. Statistical analysis was performed using a two-tailed paired *t* test. **b** Whole cell extracts were fractionated by SDS-PAGE, transferred onto PVDF membrane and immuno-blotted with the indicated antibodies. Data are from a single experiment representative of five separate experiments with similar results

In the course of this work we noted that whilst the DYRK1B^S421A^ mutant was completely defective for p-S421 in cells (Fig. 1b), ATP-competitive inhibitors of DYRK1B Ser/Thr kinase activity left some residual p-S421 signal (Fig. 1c), raising the possibility that other cellular protein kinases might contribute to S421 phosphorylation. To investigate this we co-expressed DYRK1B together with an activated KRAS^G12V^ mutant because: (i) KRAS drives activation of multiple protein kinase cascades and (ii) expression of activated KRAS has previously been proposed to activate DYRK1B by up to 20-fold [33]. Wild-type DYRK1B or kinase-dead DYRK1B^D239A^ (KD) was transiently expressed with KRAS^G12V^ for 24 h in HEK293 cells. DYRK1B proteins were then isolated from whole cell extracts by immunoprecipitation and assayed in an in vitro DYRK1B kinase assay; activity was again normalised to the amount of DYRK1B that was recovered in the immunoprecipitation (Fig. 3a). DYRK1B kinase activity was increased by co-expression with KRAS^G12V^; however, this effect was modest and not statistically significant, reflecting that fact DYRK1B was already very active when expressed in HEK293 cells. Consistent with this, KRAS expression also failed to significantly increase S421 phosphorylation over the already high basal level (Fig. 3b). As a control, DYRK1B^D239A^ (KD) exhibited negligible kinase activity that was unaffected by KRAS^G12V^ expression. Control blots confirmed that KRAS^G12V^ was expressed and active since it activated endogenous ERK1/2 (Fig. 3b). During these experiments, we observed that kinase-dead DYRK1B^D239A^ (KD), which cannot auto-phosphorylate on S421 (Fig. 1c), became phosphorylated on S421 when co-expressed with KRAS^G12V^ (Fig. 3b). This suggested that S421 could also be phosphorylated by a kinase other than DYRK1B in cells and that this kinase was activated by KRAS^G12V^.

**Fig. 4:**
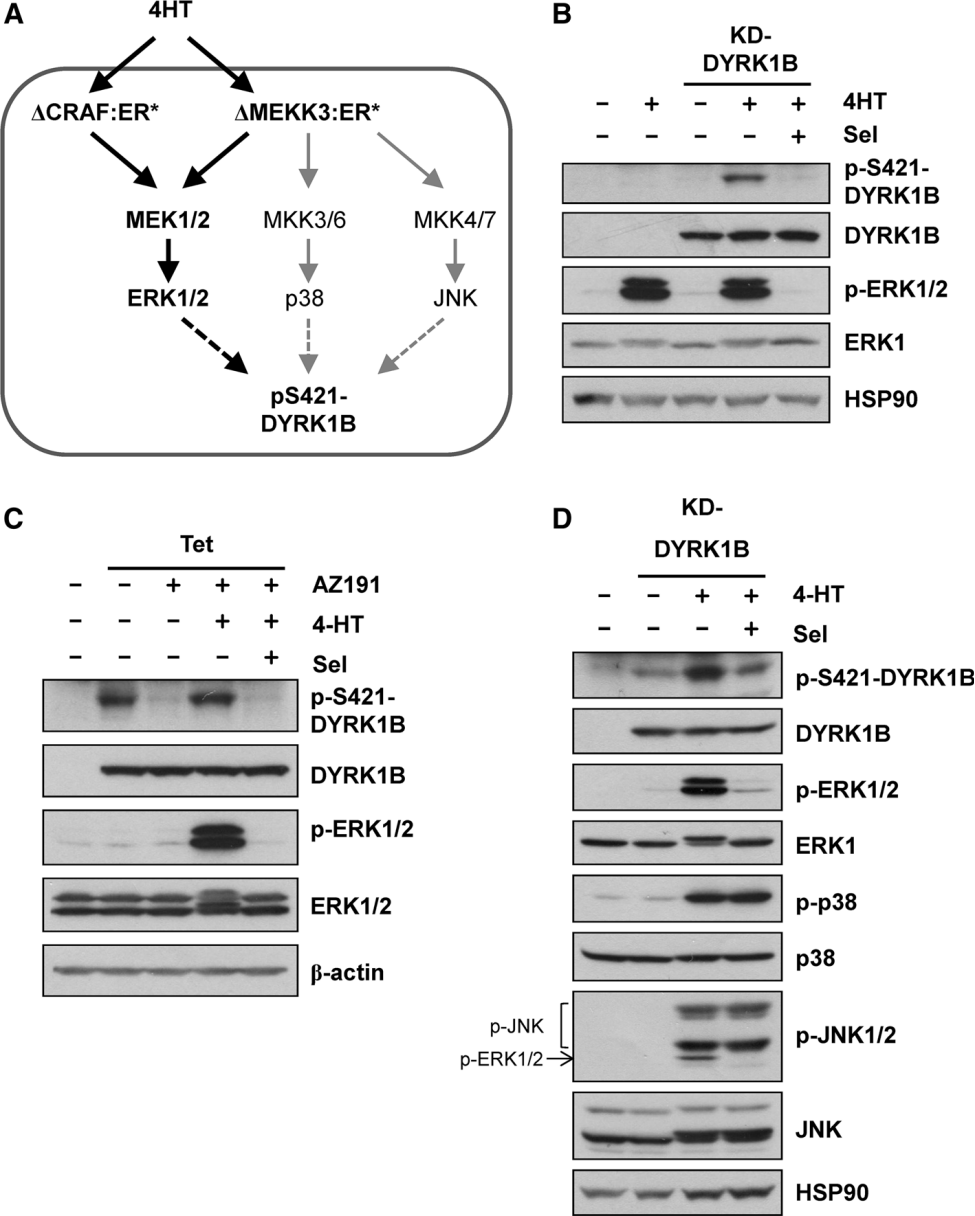
Activation of RAF-MEK1/2-ERK1/2 signalling increases S421-DYRK1B phosphorylation. **a** Schematic representation of signalling in HR1 and HM3 cells which express either the ΔCRAF:ER* or ΔMEKK3:ER* fusion proteins that are activated by 4-hydroxytamoxifen (4HT). **b** Kinase-dead DYRK1B^D239A^ (KD) was transfected into HR1 cells and ΔCRAF:ER* was activated by the addition of 100 nM 4HT in the absence or presence of 1 µM Selumetinib for a further 24 h. Whole cell lysates were fractionated by SDS-PAGE, transferred onto PVDF membrane and blotted with the indicated antibodies. **c** Wild-type DYRK1B expression was induced in HD1B cells by the addition of 1 µg ml^−1^ tetracycline in the absence or presence of 1 µM AZ191 for 24 h to reduce autophosphorylation of S421-DYRK1B. In parallel, ΔCRAF:ER* was activated by the addition of 100 nM 4-HT in the absence or presence of 1 µM Selumetinib. **d** Kinase-dead DYRK1B^D239A^ was transfected into HM3 cells and ΔMEKK3:ER* was activated by the addition of 100 nM 4-HT in the absence or presence of 1 µM Selumetinib for 24 h. Whole cell lysates were fractionated by SDS-PAGE, transferred onto PVDF membrane and blotted with the indicated antibodies. Note that the p-JNK antibody cross reacts with p-ERK1/2 that is also activated by ∆MEKK3:ER*. All data is from a single experiment representative of three separate experiments with similar results

KRAS activates multiple effector pathways including the RAF-MEK1/2-ERK1/2 pathway and various PI3K-dependent pathways. However, we noted that S421 is immediately followed by a proline residue (P422) in a Ser-Pro motif, the minimum required for phosphorylation by proline-directed kinases such as ERK1/2. To address if activation of the ERK1/2 pathway alone could increase p-S421, DYRK1B^D239A^ (KD) was expressed in HR1 cells [39]; these cells are derived from HEK293 cells and harbour the ΔCRAF:ER* fusion protein (Fig. 4a) that is activated by 4-hydroxytamoxifen (4HT). Upon activation of ΔCRAF:ER*, DYRK1B^D239A^ (KD) became phosphorylated on S421 and this was blocked by the highly selective MEK1/2 inhibitor, selumetinib (AZD6244/ARRY-142886) [40] (Fig. 4b). To investigate if S421 phosphorylation following RAF activation also occurred on wild-type DYRK1B, we used HD1B cells, a cell line that harbours ΔCRAF:ER* and exhibits tetracycline-inducible expression of DYRK1B [25]. When DYRK1B expression was induced with tetracycline, the DYRK1B protein displayed auto-phosphorylation on S421 (Fig. 4c). Treatment of these cells with the DYRK1B inhibitor AZ191 blocked p-S421 auto-phosphorylation but S421 phosphorylation was restored by ΔCRAF:ER* activation and this was again blocked by selumetinib. Thus, activation of the ERK1/2 signalling pathway was sufficient to drive p-S421 of DYRK1B in cells.

To investigate if other stress-activated, proline-directed kinases could also promote phosphorylation of S421-DYRK1B we expressed DYRK1B^D239A^ in HM3 cells; these are HEK293 cells which harbour an inducible MEKK3 fusion protein (∆MEKK3:ER*) that activates the ERK1/2, JNK and p38 pathways (Fig. 4a) [41]. Activation of ∆MEKK3:ER* with 4HT caused activation of ERK1/2, JNK1/2 and p38 and increased p-S421 on DYRK1B (Fig. 4d). However, p-S421 was again blocked by selumetinib, suggesting that S421-DYRK1B phosphorylation was mainly dependent on MEK-ERK1/2 activation and that strong activation of JNK or p38 could not compensate for loss of ERK1/2.

### ERK2 directly phosphorylates DYRK1B on S421 in cells and in vitro

**Fig. 5:**
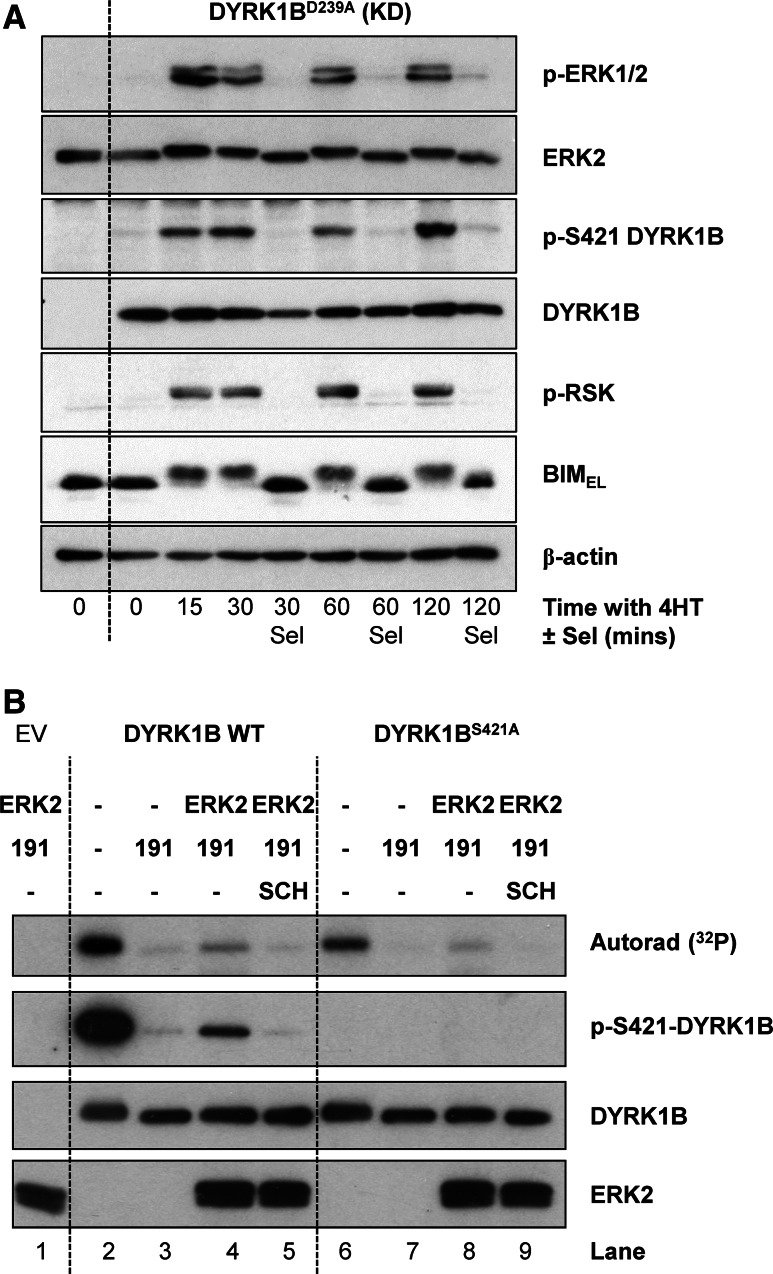
S421-DYRK1B is a direct substrate of ERK1/2 in cells and in vitro. **a** Kinase-dead DYRK1B^D239A^ (KD) was transfected into HR1 cells and ΔCRAF:ER* was activated by the addition of 100 nM 4HT in the absence or presence of 1 µM Selumetinib for 15–120 min. Whole cell lysates were fractionated by SDS-PAGE, transferred onto PVDF membrane and blotted with the indicated antibodies. **(B)** Empty vector (EV), FLAG-DYRK1B wild-type or FLAG-DYRK1B^S421A^ were transiently expressed in HEK293 cells. 4 h post-transfection, cells were treated with 10 µM AZ191 for a further 24 h to minimise DYRK1B autophosphorylation on S421. FLAG-DYRK1B proteins were then isolated from whole cell extracts by anti-FLAG immunoprecipitation and the resulting immuno-complexes were washed and used as a substrate in an ERK2 in vitro kinase assay in the presence of [γ-^32^P]ATP as described in “Materials and methods”. Some of the assay tubes included the ERK1/2 inhibitor SCH772984. The terminated assay reactions were separated by SDS-PAGE, transferred onto PVDF membrane and ^32^P incorporation was detected by autorad. Subsequently, the membrane was blotted with the indicated antibodies. Data are from a single experiment representative of two separate experiments with similar results

If DYRK1B is a direct substrate of ERK1/2 then the kinetics of S421 phosphorylation on DYRK1B should closely follow the kinetics of ERK1/2 activation in cells and should also match the phosphorylation of bona fide ERK1/2 substrates such as BIM_EL_ [42] and RSK [43]. To address this we again expressed DYRK1B^D239A^ (KD) in HR1 cells and stimulated them with 4HT for various times to activate ΔCRAF:ER*. Activation of ERK1/2 was observed as early as 15 min after 4HT addition and persisted for the full 120 min timecourse (Fig. 5a). Increases in p-S421 DYRK1B tracked precisely with p-ERK1/2, being apparent from 15 min onwards; furthermore, inclusion of selumetinib at each timepoint abolished ERK1/2 activation and the p-S421-DYRK1B signal (Fig. 5a). In addition, phosphorylation of S421 on DYRK1B precisely matched the MEK1/2-dependent phosphorylation of RSK and BIM_EL_ (monitored by it’s characteristic reduction in mobility on SDS-PAGE) following activation of ΔCRAF:ER*.

To determine if ERK2 could directly phosphorylate DYRK1B at S421, recombinant, active GST-ERK2 was incubated with FLAG-DYRK1B in an in vitro kinase assay (Fig. 5b). In this assay, wild-type DYRK1B was able to auto-phosphorylate on S421 in the absence of AZ191 (Lane 2) as shown by ^32^P incorporation and pS421-DYRK1B western blot. However, ^32^P was still incorporated into DYRK1B^S421A^ in the absence of AZ191 (Lane 6), albeit to a lesser extent than wild-type DYRK1B, suggesting that there are other sites of DYRK1B auto-phosphorylation in vitro in addition to S421, consistent with our MS analysis (Table 1, Supplementary Table 1). In the presence of AZ191, DYRK1B auto-phosphorylation was blocked (Lanes 3 and 7). When active ERK2 was added to DYRK1B in the presence of AZ191, DYRK1B became phosphorylated on S421 (Lane 4) as shown by ^32^P incorporation and p-S421 western blot and this was blocked by the ERK1/2 inhibitor SCH772984 [44] (Lane 5) demonstrating that S421-DYRK1B was a direct substrate of ERK2 in vitro. Interestingly, ERK2 was also able to phosphorylate DYRK1B^S421A^ (Lane 8) although to a lesser degree, indicating that ERK2 may also be able to phosphorylate other sites on DYRK1B in vitro. Taken together these results demonstrated that in addition to auto-phosphorylation, DYRK1B is also phosphorylated on S421 by ERK1/2 in cells and in vitro.

### Inhibition of MEK1/2-ERK1/2 signalling by selumetinib promotes DYRK1B expression

**Fig. 6:**
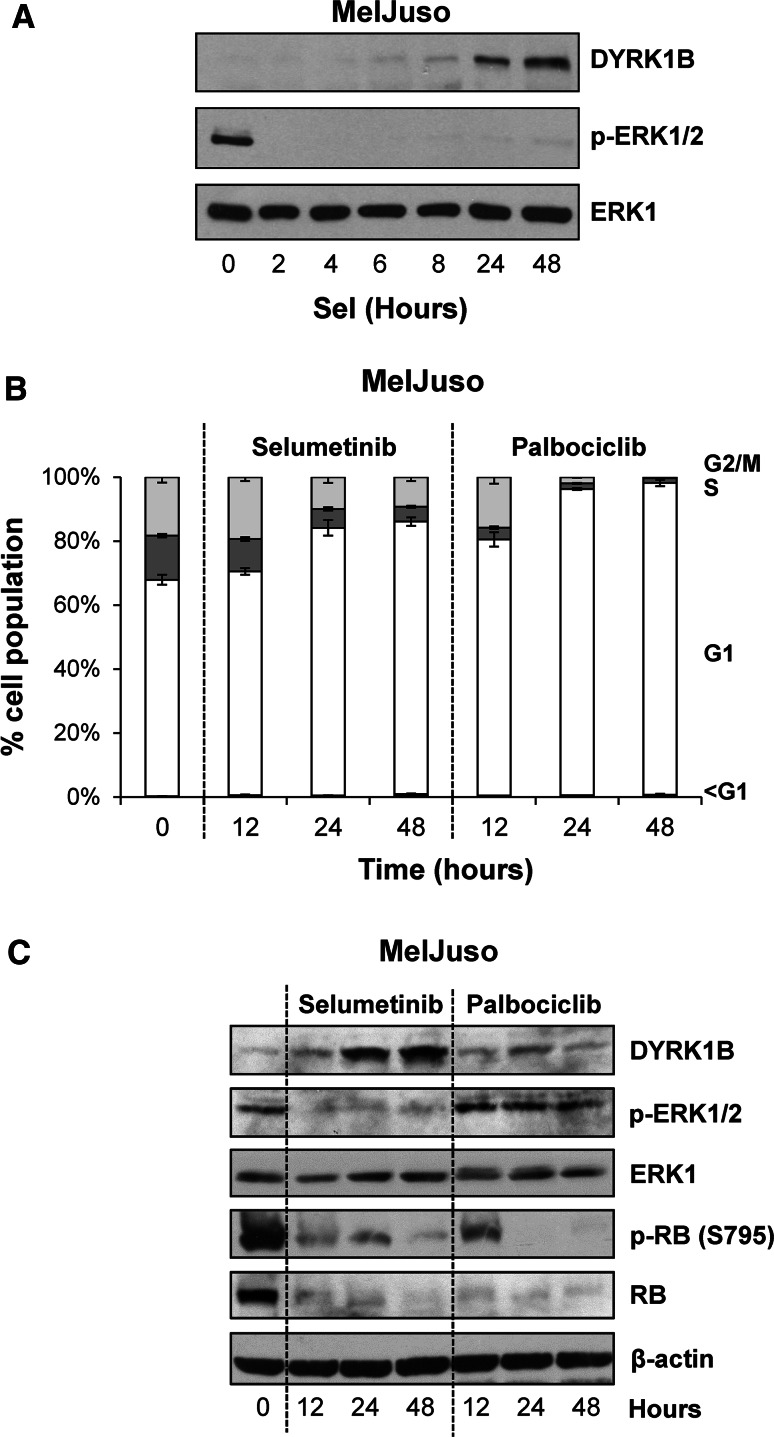
Inhibition of ERK1/2 signalling increases DYRK1B expression. **a** MelJuso cells were treated with 1 µM selumetinib for 2–48 h. Whole cell extracts were fractionated by SDS-PAGE, transferred onto PVDF membrane and immuno-blotted with the indicated antibodies. **b**, **c** MelJuso cells were treated for 12–48 h with 1 µM Selumetinib or 5 µM Palbociclib and the cells were fixed, stained with propidium iodide and cell cycle distribution was determined by flow cytometry **(b)** and whole cell extracts were fractionated by SDS-PAGE, transferred onto PVDF membrane and immuno-blotted with the indicated antibodies **(c)**. Data is from a single experiment representative of three separate experiments with similar results. The data in (**b**) are mean ± S.D. from a single experiment with three replicate dishes of cell per data point; similar results were obtained in two additional experiments

It has previously been show that first generation, pan-MEK1/2/5 inhibitors PD98059 and U0126 can increase DYRK1B mRNA and protein [22, 45]. Notwithstanding the off-target effects of these drugs these results suggest that ERK1/2 signalling may normally repress DYRK1B expression. Our demonstration that ERK1/2 phosphorylates DYRK1B at S421 prompted us to examine DYRK1B expression using the more selective, second generation MEK1/2 inhibitor selumetinib [40]. Indeed, when we treated the melanoma cell line MelJuso (HRAS^G13D^/NRAS^Q61L^) with 1 µM selumetinib this strongly and immediately inhibited ERK1/2 signalling and increased the expression of DYRK1B after a delay of 8 h. This was accompanied by an increase in DYRK1B mRNA which was apparent after 5 h of selumetinib treatment (Supplemental Fig. 1a). Treatment with actinomycin D, an inhibitor of transcription, prevented the increase in DYRK1B expression observed in response to selumetinib treatment (Supplemental Fig. 1b) suggesting that the increase in DYRK1B expression observed upon MEK inhibition reflects de novo gene transcription. Finally, similar results were obtained in the A375 melanoma cell line (BRAF^V600E^) (Supplemental Fig. 1c), where selumetinib caused a dose-dependent inhibition of ERK1/2 signalling and an increase in expression of DYRK1B as well as BIM_EL_, which is known to be repressed by the ERK1/2 pathway [46]. Since inhibition of ERK1/2 signalling by selumetinib typically causes a G1 cell cycle arrest [47] (Fig. 6b), the increase in DYRK1B expression could simply be a consequence of cell cycle arrest rather than a specific consequence of reduced ERK1/2 signalling. To address this, we compared the effect of selumetinib with that of palbociclib (PD-0332991), a potent and highly selective inhibitor of CDK4 and CDK6 [48]. Whilst both drugs elicited a strong G1 cell cycle arrest (Fig. 6b) and inhibited phosphorylation of RB (Fig. 6c), only selumetinib inhibited ERK1/2 signalling and increased DYRK1B expression (Fig. 6c), demonstrating that the increase in DYRK1B was not simply a consequence of cell cycle arrest. Thus ERK1/2 signalling provides opposing regulatory inputs into DYRK1B: ERK1/2 can directly phosphorylate DYRK1B at S421, a residue that promotes kinase activity, but ERK1/2 signalling also represses DYRK1B expression.

**Fig. 7:**
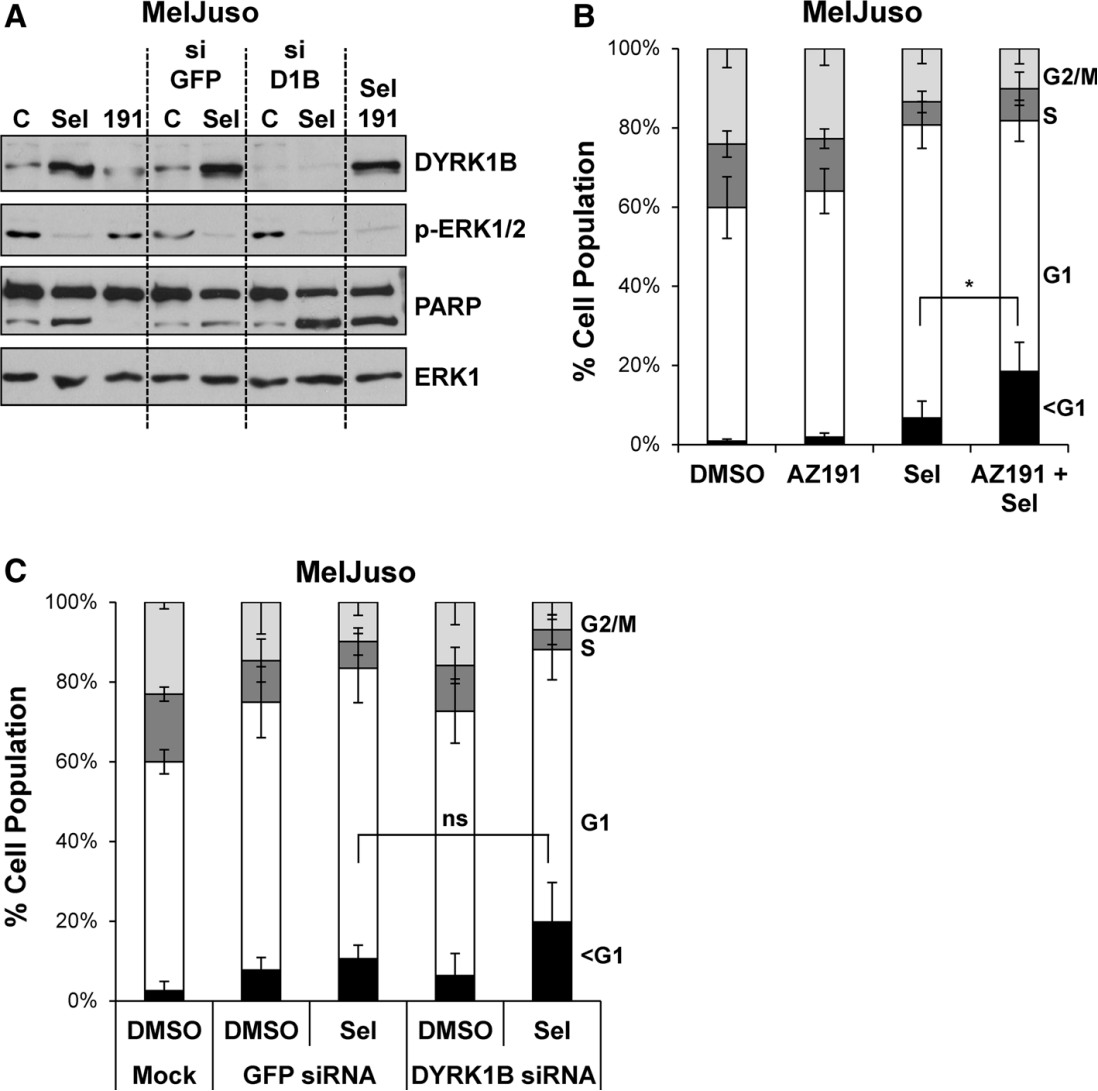
Dual inhibition of ERK1/2 and DYRK1B is not synthetic lethal. MelJuso cells were transfected with DYRK1B or GFP siRNA prior to treatment with DMSO, 1 µM selumetinib or 10 µM AZ191 for 48 h. **a** Whole cell extracts were fractionated by SDS-PAGE, transferred onto PVDF membrane and immuno-blotted with the indicated antibodies to confirm inhibition of ERK1/2 signalling and knockdown of DYRK1B. **b** MelJuso cells treated for 48 h with AZ191, selumetinib or the combination were fixed, stained with propidium iodide and cell cycle distribution determined by flow cytometry. **c** MelJuso cells treated for 48 h with DYRK1B siRNA, selumetinib or the combination were fixed, stained with propidium iodide and cell cycle distribution determined by flow cytometry. In **d**, **e** data represents mean ± S.D. from four biological replicate experiments, each with three technical replicates. Statistics were performed on the population of cells with sub-G1 DNA using one-way ANOVA, with* asterisk* denoting *p* = 0.017 and ns (not significant) denoting *p* = 0.167

DYRK1B has been proposed to function as a pro-survival protein kinase [30–32]. Since ERK1/2 inhibition by selumetinib typically results in a G1 cell cycle arrest with little cell death [47] we considered the possibility that DYRK1B expression might provide a survival signal following MEK1/2-ERK1/2 inhibition, thereby preventing cell death; that is, combined inhibition of MEK1/2 and DYRK1B would be synthetic lethal. To test this we treated MelJuso cells with selumetinib alone or in combination with the DYRK1B inhibitor AZ191 or in combination with DYRK1B siRNA, with GFP siRNA as a control (Fig. 7a). AZ191 treatment alone had no effect on cell death as judged by PARP cleavage (Fig. 7a) and the fraction cells with hypodiploid (sub-G1) DNA (Fig. 7b). Selumetinib treatment caused only a small increase in PARP cleavage and cell death. The combination of AZ191 and selumetinib caused greater cell death than either drug alone; however, whilst this was statistically significiant, it was only a very modest effect. We also observed a modest increase in cell death when we combined selumetinib with DYRK1B siRNA; however, this was not statistically significant (Fig. 7c). Finally, despite the induction of DYRK1B, we failed to observe any increase in cell death when selumetinib was combined with AZ191 or DYRK1B siRNA in A375 cells (data not shown). Thus, any synthetic lethal relationship between DYRK1B and MEK1/2 was at best weak and variable between cell lines.

### Mutants of DYRK1B found in cancer or metabolic syndrome exhibit normal or reduced kinase activity

**Fig. 8:**
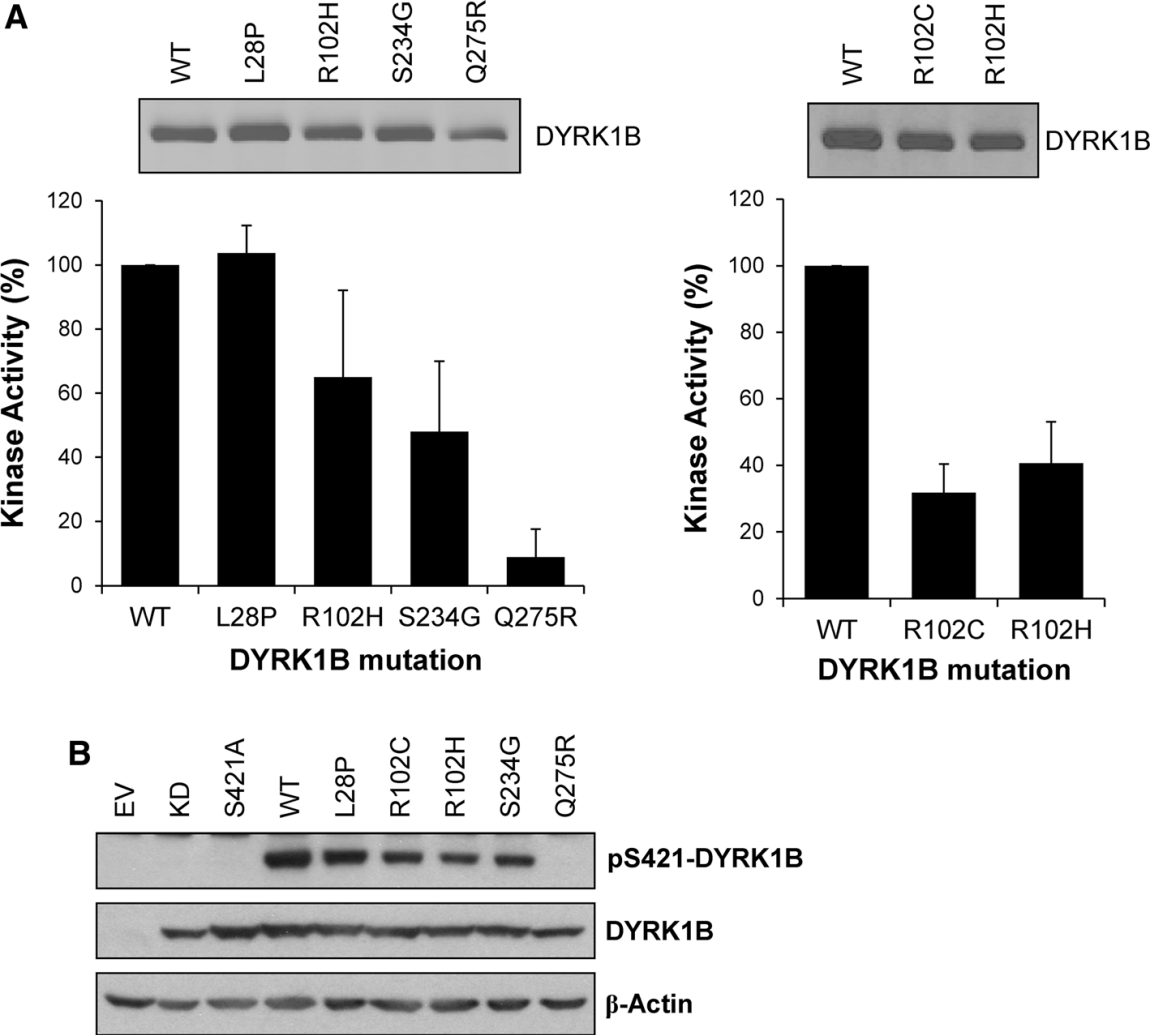
DYRK1B mutants found in cancer and metabolic syndrome exhibit reduced intrinsic kinase activity. **a** Wild-type DYRK1B or the indicated mutant DYRK1B constructs were transiently expressed for 24 h in HEK293 cells. FLAG-DYRK1B proteins were isolated from whole cell extracts by immunoprecipitation and immuno-complexes were divided into two aliquots. The first aliquot was separated by SDS-PAGE, transferred onto PVDF membrane and immunoblotted for DYRK1B (*upper panels*). DYRK1B levels were quantified on Licor Odyssey. The second aliquot was incubated with the synthetic DYRK substrate peptide Woodtide and 0.1 mM [γ-^32^P]ATP in an in vitro kinase assay to determine kinase activity as described in materials and methods (*lower panel*). In vitro kinase activity was normalised to the amount of each DYRK1B protein present on the immuno-blot. Data are shown as mean ± standard deviation from three separate experiments each with three technical replicates. **(b)** Wild-type DYRK1B or the indicated mutant DYRK1B constructs were transiently expressed for 24 h in HEK293 cells. Whole cell extracts were separated by SDS-PAGE, transferred onto PVDF membrane and immuno-blotted with the antibodies indicated. Data is from a single experiment representative of three separate experiments with similar results

Systematic sequencing of cancer genomes has revealed rare mutations in DYRK1B including L28P, R102H, S234G and Q275R [29]. However, the low incidence of these mutations calls into question whether they are ‘drivers’ or ‘passengers’ and no further functional information has been forthcoming. In addition, mutations in DYRK1B have been found in familial cases of metabolic syndrome [26, 49] and include: gain-of-function mutations R102C and H90P; putative loss-of-function mutations, such as L28P, and apparently benign mutations, such as G352A and P578S. However, the impact of these disease-associated mutations on intrinsic kinase activity has not been described. To address this directly we engineered some of these mutations into individual DYRK1B constructs, expressed them in HEK293 cells, isolated them by immunoprecipitation and assayed their kinase activity in vitro. Our results revealed a consistent pattern: DYRK1B^L28P^ (found in rare cases of metabolic syndrome as well as cancer) exhibited identical kinase activity to wild-type DYRK1B; DYRK1B^R102H^ and DYRK1B^S234G^ exhibited 40–50 % reduction in kinase activity and DYRK1B^Q275R^ exhibited a 90 % reduction in kinase activity (Fig. 8a). We also directly compared the activity of the R102H mutant (found in cancer) and the R102C mutant (found in metabolic syndrome); both mutants exhibited a 50–60 % reduction in kinase activity (Fig. 8a). Finally, the relative activity of these DYRK1B mutants correlated well with their degree of phosphorylation at S421 (Fig. 8b) suggesting that in the absence of strong inputs from the ERK1/2 pathway p-S421 correlated well with intrinsic activity. Thus mutations in DYRK1B that are found in cancer (L28P, R102H, S234G and Q275R) or metabolic syndrome (L28P, R102C) have either no effect on intrinsic kinase activity or reduce kinase activity, in one case (DYRK1B^Q275R^) severely.

## Discussion

### *Trans*-autophosphorylation at S421 contributes to DYRK1B activity

Our results identified S421 as a site of DYRK1B autophosphorylation since it was present in purified recombinant wild-type DYRK1B incubated in vitro with ATP, absent in kinase-dead DYRK1B and strongly (though not completely) inhibited by DYRK1B inhibitors in vitro or in cells. These drugs do not prevent autophosphorylation on tyrosine (largely Y273, but also Y63) in cells [25] except at high concentrations [36], consistent with phosphorylation of Y273 and S421 proceeding through different mechanisms. Indeed, we have now shown that S421 autophosphorylation can proceed through an intermolecular *trans*-phosphorylation mechanism whereas Y273 is an intramolecular *cis*-phosphorylation event. To our knowledge, this is the first description of a serine or threonine auto-phosphorylation site on DYRK1B. S520 in DYRK1A was previously identified as a site of auto-phosphorylation that was required for full DYRK1A kinase activity [18]; in this case S520 auto-phosphorylation increased 14-3-3β binding to DYRK1A. However, phosphorylation of S520 in DYRK1A took place in *cis*, in contrast to the intermolecular phosphorylation of S421 in DYRK1B described here. Indeed, given the position of S421, situated away from the kinase active site, it is difficult to imagine it could be phosphorylated in *cis* through an intramolecular mechanism. Regardless, it seems that S421 phosphorylation also contributes to DYRK1B kinase activity, either directly or by an indirect mechanism involving accessory proteins, since the S421A mutant exhibited a consistent 50 % reduction in kinase activity; indeed, this is likely to be an underestimate of the impact of the S421A mutant since only half of over-expressed DYRK1B was phosphorylated at S421. Whilst a twofold modulation of kinase activity may seem modest, it is important to remember that even a 50 % increase in expression and activity of the closely related DYRK1A has profound physiological effects in mice and phenocopies some Down’s syndrome defects [11, 15] whilst a 50 % decrease in DYRK1A causes even more dramatic effects in *Drosophila,* mice and human patients including microcephaly, growth retardation and behavioural defects [11, 50, 51].

S421 occurs close to the highly conserved arginine residue (AA**R**I**S** ^**421**^PL) shortly after alpha helix H/kinase sub-domain XI [3, 10]. Serine and threonine are the first and third most common residues at this position across the kinome as a whole, whilst the Cancer Genome Atlas (TCGA) reveals that the position equivalent to S421 is ranked 25^th^ in terms of frequency out of the 2500 identified mutation positions across kinase domains, suggesting a potential wider significance for phosphorylation at this site. Of the 61 human CMGC kinases listed at http://kinase.com/human/kinome/groups/cmgc.aln only 7 lack a Ser or Thr at the position corresponding to Ser421 of DYRK1B. More significantly, S421 in DYRK1B is a Pro-directed site (Ser-Pro) and within the closely related human DYRK family (DYRK1A, 1B, 2, 3 & 4 and the four HIPKs) the corresponding site is a Ser-Pro or Thr-Pro in all cases except DYRK1A (Gln-Pro) raising the possibility that these kinases may also be regulated by phosphorylation at this site. This site is also Ser-Pro or Thr-Pro in DYRKs from lower eukaryotes including *S. pombe* Pom1p, *S. cerevisiae* Yak1p, *C. elegans* MBK-1 and MBK-2 and *D. melanogaster* dDYRK2 and dDYRK3, indicating evolutionary conservation of the site. In terms of functional relevance, intermolecular phosphorylation at S421 suggests that DYRK1B may be able to form dimers. Indeed, the structure of the closely related DYRK1A [10] revealed multiple copies per unit cell with Q469 of DYRK1A, corresponding to S421 of DYRK1B, well away from the ATP binding pocket at the interface between DYRK1A dimers. It is currently unclear if DYRK1B is a dimer in cells or whether it is more or less active as a dimer or monomer but it will be interesting to determine if S421 phosphorylation influences dimerisation. Alternatively, S421 phosphorylation may influence substrate interactions, which would be consistent with the reduction in kinase activity that we observe for the S421A mutant. For example, the closely related DYRK4 exhibits an alternative splicing donor site in exon 19 which removes three amino acids (∆CLV) at the end of the α-helix H, immediately prior to the conserved Arg in subdomain XI [21]. This region is thought to contribute to the substrate-binding structure [52] and it is notable that DYRK4^ΔCLV^ exhibits reduced kinase activity against exogenous substrates compared to the full length DYRK4 [21]. Thus, it is possible that changes to this region in DYRK1B, such as phosphorylation of S421, might contribute to ordering of the substrate-binding structure. Such speculation will need to be addressed by future structural studies.

### ERK1/2 can phosphorylate DYRK1B at S421

We sought to identify DYRK1B autophosphorylation sites as biomarkers for DYRK1B kinase activity and identified S421 as a phosphorylation site that promotes DYRK1B activity. However, we found that S421 is also phosphorylated by ERK1/2; whilst this disqualifies S421 as a unique DYRK1B activity biomarker it reveals a new mode of cross talk between ERK1/2 and DYRK1B.

We noted that inhibitors of DYRK1B Ser/Thr kinase activity left a residual p-S421 signal in cells, prompting a search for other kinases that might target this site. For this purpose we co-expressed DYRK1B with KRAS^G12V^, since KRAS has several effectors (PI3K, RAL, etc.,) and activates multiple protein kinase signalling pathways. In contrast to previous reports of a 9- to 20-fold activation [33] we consistently found that KRAS^G12V^ caused only a very modest (10–15 %) increase in activity that was not statistically significant. This was not due to a deficit in our KRAS construct as KRAS^G12V^ strongly activated endogenous ERK1/2 in the same transfections. These results suggest that any effect of activated KRAS on DYRK1B effector pathways such as Hedgehog [34] may be independent of DYRK1B intrinsic kinase activity, though we cannot rule out a possible effect on DYRK1B substrate binding (see above) that might be missed in an assay that employs simplified peptide substrates. However, these experiments also revealed that KRAS^G12V^ could actually increase DYRK1B phosphorylation at S421. Through a combination of conditional kinases and selective kinase inhibitors we were able to show that ERK1/2 activation alone could increase p-S421, commensurate with established ERK1/2 substrates, and ERK2 could phosphorylate S421 in vitro. This is the first report to clearly define DYRK1B as a substrate of ERK2. A previous study suggested that ERK2, JNK or p38 might phosphorylate DYRK1B but stopped short of specifying the kinase, identifying the phosphorylation site or the effect on DYRK1B function [53].

Together with previous studies, our results reveal a complex interplay between ERK1/2 signalling and DYRK1B. Since phosphorylation of S421 may enhance DYRK1B kinase activity, ERK1/2 inhibition could decrease DYRK1B activity. In contrast, previous studies have indicated that inhibition of ERK1/2 signalling may increase DYRK1B expression [22, 45] and we also observed this with the highly selective MEK1/2 inhibitor selumetinib, suggesting that ERK1/2 signalling can repress DYRK1B expression. Thus, ERK1/2 inhibition may reduce the activity of existing DYRK1B but also increase de novo DYRK1B expression, providing a mechanism for fine tuning DYRK1B activity. It is well known that the ERK1/2 pathway is a key regulator of the cell cycle, promoting proliferation or cell cycle arrest depending on the magnitude or duration of ERK1/2 activity [54]. DYRK1A and DYRK1B have also emerged as regulators of the cell cycle; both can promote the turnover of CCND1, increase expression of p21^CIP1^ and/or p27^KIP1^ and thereby promote cell cycle arrest and differentiation [22, 24, 25, 45, 55]. Indeed, even modest (~50 %) changes in DYRK1A expression and activity exert profound effects on the cell cycle [56]. ERK1/2-dependent regulation of DYRK1B adds another level of control, providing a link between two cell fate signalling pathways.

There is growing interest in identifying synthetic lethal drug combinations in the treatment of cancer [57]. For example, whilst selumetinib exerts a largely cytostatic effect, combining the BH3-mimetic ABT-263 with selumetinib causes up to 80 % cell death in colorectal cells and delays the onset of acquired resistance to selumetinib [47]. The increase in DYRK1B expression following MEK1/2-ERK1/2 inhibition prompted us to investigate whether inhibition of DYRK1B might similarly transform the cytostatic effects of selumetinib to promote cell death. Indeed, Gao et al. recently described a twofold increase in H292 cell death when the pan-MEK1/2/5 inhibitor U1026 was combined with DYRK1B siRNA [45], although they did not report the actual percentage of dead cells. We also observed a twofold increase in cell death in MelJuso cells when selumetinib was combined with DYRK1B siRNA or the AZ191 DYRK1B inhibitor. However, the percentage of dead cells observed under these conditions was modest, being less than 20 %, and was not observed in a second cell line (A375) that exhibited strong DYRK1B upregulation following ERK1/2 inhibition. Thus, DYRK1B and MEK1/2 do not seem to exhibit a robust synthetic lethal relationship that might form the basis for a rational drug combination.

### DYRK1B mutants found in metabolic syndrome or cancer exhibit normal or reduced kinase activity

DYRK1B is over-expressed due to gene amplification in certain tumours, including pancreatic cancer [27, 28], and this has led to the suggestion that DYRK1B is a potential oncogene. In addition, cancer genome sequencing has revealed mutations in DYRK1B including L28P, R102H, S234G and Q275R [29]. Both L28 and R102 lie outside the DYRK1B kinase domain; L28 lies outside any known conserved regions whilst R102 is situated between the DYRK homology (DH) box and the kinase domain. S234 is conserved in the class 1 DYRKs and immediately precedes the catalytic loop in kinase subdomain VIb whilst Q275 is conserved in all DYRKs and follows the critical Y273 in the kinase activation loop within subdomain VIII. In addition, L28P and R102C mutations were found in cases of metabolic syndrome [26, 49]. However, these disease-associated mutations were found at low frequency and their impact on DYRK1B activity was not assessed.

We found that these mutations were either neutral with respect to DYRK1B activity (L28P) or caused a consistent reduction in kinase activity (R102H, R102C S234G and Q275R); in the case of the Q275R mutation this effect was severe, with an approximate 90 % reduction in kinase activity. Q275 lies within the activation loop of DYRK1B; whilst activating, oncogenic mutations are found in this region at significant frequency in some protein kinases, mutations in this loop, including the introduction of bulky and highly positive side chain such as arginine, are also known to inhibit activity by impacting on activation loop conformation and flexibility. At this stage we cannot rule out the possibility that these mutations might confer kinase-independent properties upon DYRK1B; for example, by driving DYRK1B to a specific cellular location or engaging with particular partner proteins. Indeed, some of the effects of DYRK1B in metabolic syndrome were apparently independent of DYRK1B kinase activity [26]. Regardless, our results suggest that these DYRK1B mutations are unlikely to be gain-of-function in terms of intrinsic kinase activity and some may even be loss-of-function mutations. Since DYRK1B can promote cell cycle arrest and differentiation [22, 24, 25, 45, 55, 56], such loss-of-function mutations suggest that any oncogenic properties of DYRK1B are, at best, context-dependent; indeed, they may even suggest tumour suppressor functions for wild-type DYRK1B.

In summary, we demonstrate that phosphorylation of S421 contributes to DYRK1B kinase activity and that S421 is a site for DYRK1B autophosphorylation and phosphorylation by ERK1/2, defining DYRK1B as a new substrate of ERK1/2. Our results reveal opposing effects of ERK1/2 signalling on DYRK1B which may serve to fine tune DYRK1B activity. However, despite the ability of ERK1/2 to phosphorylate S421, KRAS^G12V^ does not increase DYRK1B kinase activity. This may be because DYRK1B expressed in cells is already active so that any effects of KRAS are modest and incremental; this may require single cell analysis to resolve. Alternatively, different KRAS effectors may exert opposing effects on DYRK1B; so whilst activation of RAF-MEK-ERK may promote S421 phosphorylation, that would enhance activity, other KRAS effector pathways may exert opposing effects on DYRK1B. Indeed, our mass spectrometry analysis has identified at least two other phospho-Ser sites on DYRK1B expressed in cells that are not autophosphorylation sites; the kinases for these sites and the functional consequences remain unknown. Finally, mutants of DYRK1B found in cancer or metabolic syndrome are either neutral with respect to kinase activity or exhibit significant decreases in kinase activity. At least one of these, R102C in metabolic syndrome, is defined as a gain-of-function mutation [26] but clearly exhibits reduced kinase activity, raising the possibility that some effects of DYRK1B may not be strictly dependent on kinase activity.

### Electronic supplementary material

Below is the link to the electronic supplementary material.

Supplementary material 1 Specific inhibition of MEK-1/2-ERK1/2 signalling increases DYRK1B mRNA levels and require new gene transcription. **(A)** MelJuso cells were incubated with 1 µM Selumetinib for the time points indicated. Cells were lysed in TRIzol, RNA was extracted and cDNA was generated by reverse transcription. The cDNA was used as the template in a QPCR reaction using SyBr green PCR master mix with DYRK1B or B2 M primers. The level of DYRK1B expression was normalised to that of the housekeeping gene B2 M. **(B)** MelJuso cells were treated with 0 or 1 µM Selumetinib in the absence or presence of 10 µM Actinomycin D for 16 h. Whole cell extracts were separated by SDS-PAGE, transferred onto PVDF membrane and immunoblotted with the specified antibodies. Data are taken from a single experiment representative of three separate experiments. **(C)** A375 cells were treated for 24 h with the indicated doses of selumetinib. Whole cell extracts were fractionated by SDS-PAGE, transferred onto PVDF membrane and immuno-blotted with the indicated antibodies. Data are taken from a single experiment representative of two separate experiments

Supplementary material 2 Sites of DYRK1B autophosphorylation identified in vitro. Recombinant DYRK1B was allowed to autophosphorylate in the presence of ATP. Coomassie-stained bands of (0.5 µg or 1 µg) were reduced, cysteines blocked, and digested with: trypsin only; AspN and trypsin; chymotrypsin only; chymotrypsin and trypsin. The resultant peptides were analysed using the LCMSMS workflow on the QSTAR Elite mass spectrometer. Peptides and phosphorylation sites were identified using Mascot. *Precise location of single phosphorylation site not known

## Abbreviations

ATM: Ataxia telangiectasia mutated kinase
BRAF: v-raf murine sarcoma viral oncogene homologue B1
CCND1: Cyclin D1
CDK: Cyclin-dependent kinase
CRAF: v-raf-1 murine leukaemia viral oncogene homologue 1
DYRK: Dual-specificity tyrosine-phosphorylation-regulated kinase
EGFP: Enhanced green fluorescent protein
ERK: Extracellular signal-regulated kinase
HRAS: v-Ha-ras Harvey rat sarcoma viral oncogene homologue
JNK: c-Jun N-terminal kinase
KRAS: v-Ki-ras2 Kirsten rat sarcoma viral oncogene homologue
MEK: Mitogen-activated protein kinase/ERK kinase
MEKK: MEK Kinase
NRAS: Neuroblastoma RAS viral (v-ras) oncogene homologue
p21^CIP1^: p21 CDK-interacting protein 1
p27^KIP1^: p27 kinase inhibitory protein 1
p38: p38 kinase
PARP: Poly-ADP ribose polymerase
PI3K: Phosphoinositide 3-kinase
