# Supplementary figures and images for: Identification of DYRK1B as a substrate of ERK1/2 and characterisation of the kinase activity of DYRK1B mutants from cancer and metabolic syndrome
Source: Cell Mol Life Sci. 2015 Sep 7;73(4):883–900. doi: 10.1007/s00018-015-2032-x (PMC4735261; doi:10.1007/s00018-015-2032-x)

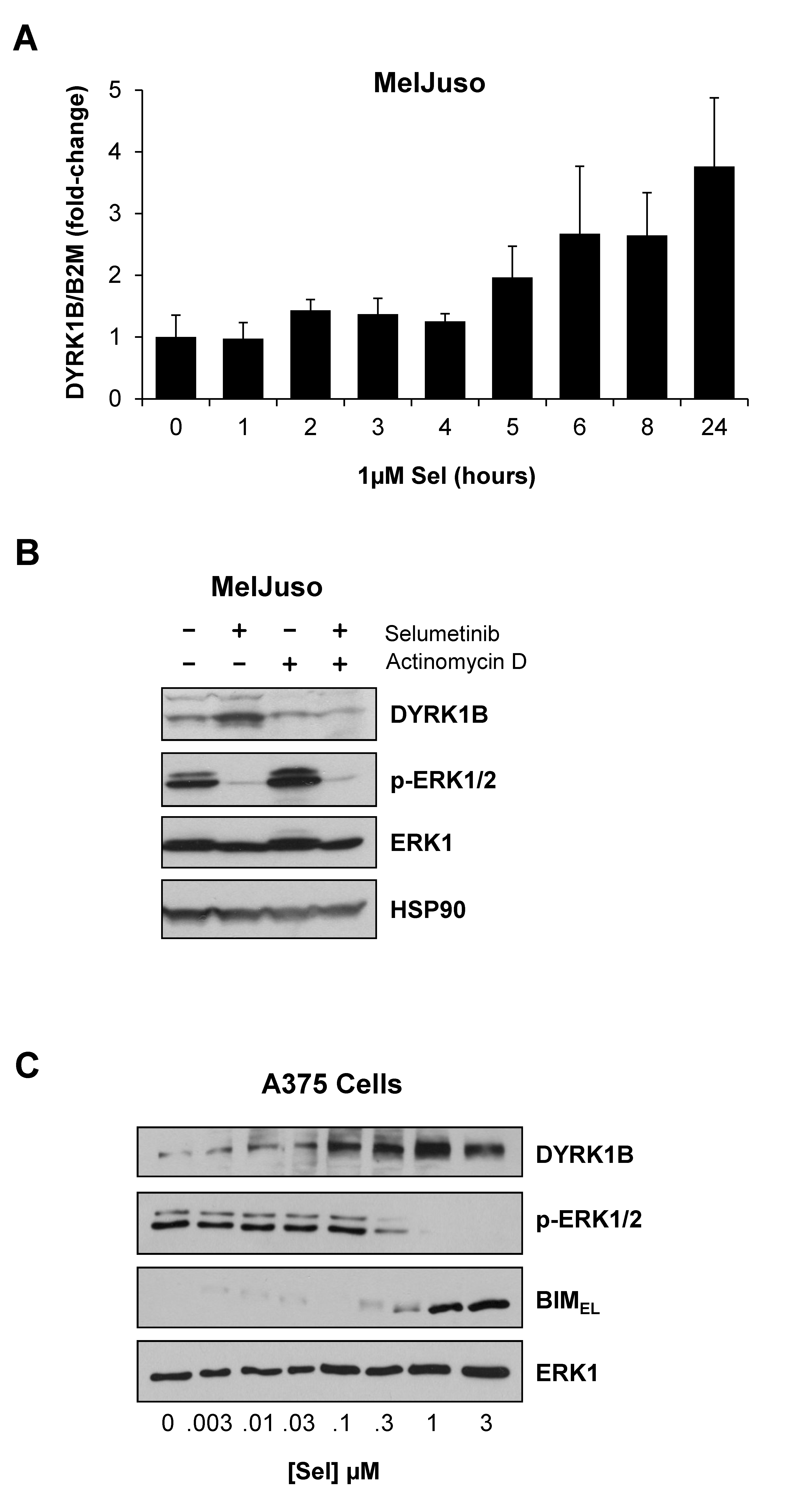

Supplement: Supplementary file 1 — Supplementary material 1 Specific inhibition of MEK-1/2-ERK1/2 signalling increases DYRK1B mRNA levels and require new gene transcription. (A) MelJuso cells were incubated with 1 µM Selumetinib for the time points indicated. Cells were lysed in TRIzol, RNA was extracted and cDNA was generated by reverse transcription. The cDNA was used as the template in a QPCR reaction using SyBr green PCR master mix with DYRK1B or B2 M primers. The level of DYRK1B expression was normalised to that of the housekeeping gene B2 M. (B) MelJuso cells were treated with 0 or 1 µM Selumetinib in the absence or presence of 10 µM Actinomycin D for 16 h. Whole cell extracts were separated by SDS-PAGE, transferred onto PVDF membrane and immunoblotted with the specified antibodies. Data are taken from a single experiment representative of three separate experiments. (C) A375 cells were treated for 24 h with the indicated doses of selumetinib. Whole cell extracts were fractionated by SDS-PAGE, transferred onto PVDF membrane and immuno-blotted with the indicated antibodies. Data are taken from a single experiment representative of two separate experiments [file 18_2015_2032_MOESM1_ESM.tiff]

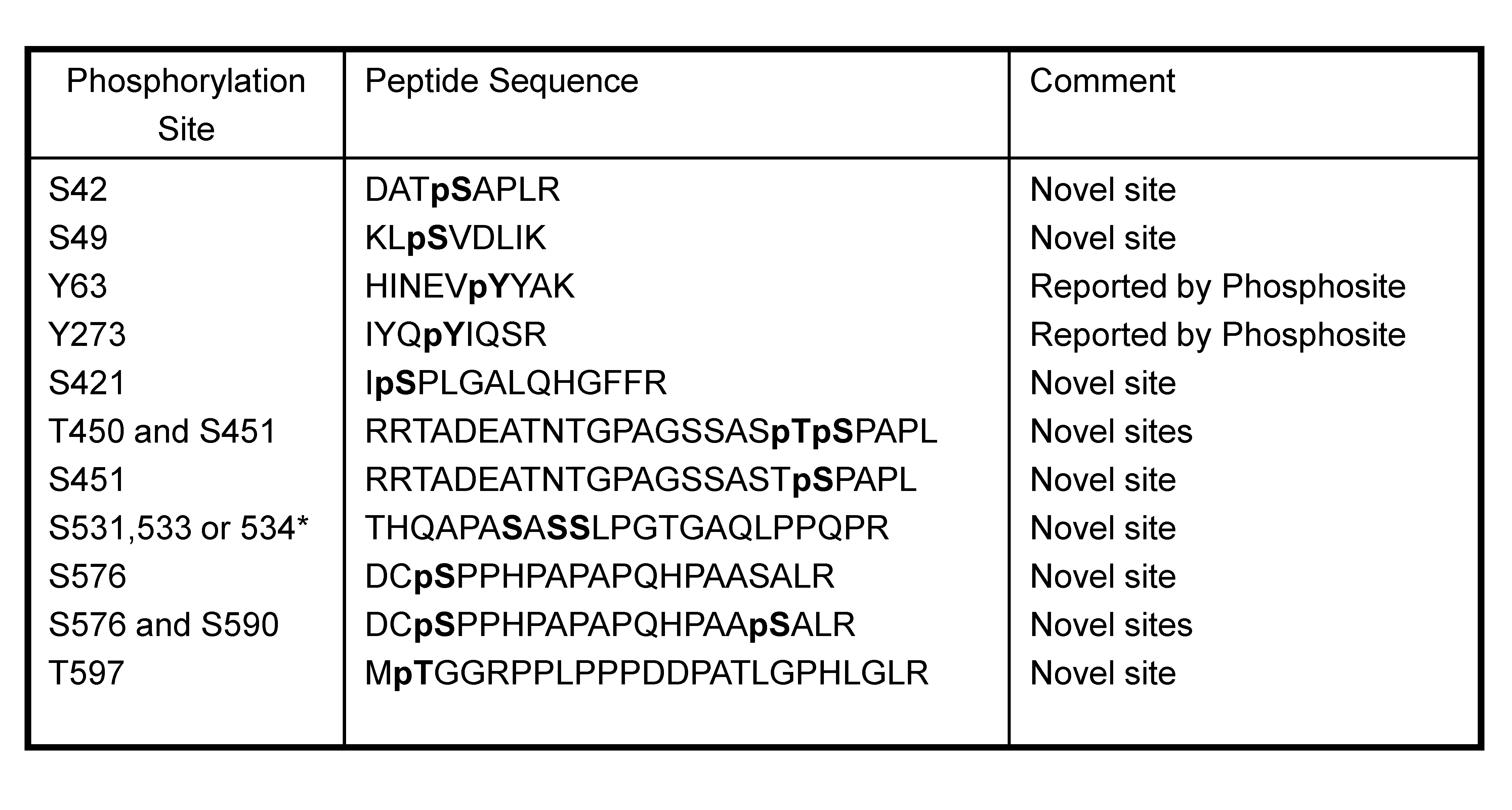

Supplement: Supplementary file 2 — Supplementary material 2 Sites of DYRK1B autophosphorylation identified in vitro. Recombinant DYRK1B was allowed to autophosphorylate in the presence of ATP. Coomassie-stained bands of (0.5 µg or 1 µg) were reduced, cysteines blocked, and digested with: trypsin only; AspN and trypsin; chymotrypsin only; chymotrypsin and trypsin. The resultant peptides were analysed using the LCMSMS workflow on the QSTAR Elite mass spectrometer. Peptides and phosphorylation sites were identified using Mascot. *Precise location of single phosphorylation site not known [file 18_2015_2032_MOESM2_ESM.tiff]
